# Supplementary material for: Cortical Response to Acute Implantation of the Utah Optrode Array in Macaque Cortex
Source: Adv Healthc Mater. 2025 Sep 15;15(3):e00575. doi: 10.1002/adhm.202500575 (PMC12817112; doi:10.1002/adhm.202500575)
Supplement: Supplementary file 1 — Supporting Information [file ADHM-15-0-s001.pdf]

ADVANCED HEALTHCARE MATERIALS

Supporting Information

for *Adv. Healthcare Mater.*, DOI 10.1002/adhm.202500575

Cortical Response to Acute Implantation of the Utah Optrode Array in Macaque Cortex

Adrián Villamarin-Ortiz, Christopher F. Reiche, Frederick Federer, Andrew M. Clark, John D. Rolston, Cristina Soto-Sánchez, Eduardo Fernandez, Steve Blair and Alessandra Angelucci**

SUPPORTING INFORMATION

SUPPORTING FIGURE LEGENDS

Supporting Fig. 1. UOA insertion procedure and device.

(A) Cartoon illustration of the UOA insertion procedure. The UOA is placed on the pia mater at the desired implantation site. The pneumatic inserter ("inserter", Blackrock Neurotech, USA) is attached to a conventional stereotaxic manipulator arm (Kopf Instruments, USA) and positioned above the UOA backplane. **(B)** Schematic overview of the main components of the inserter. When the implantation trigger is pushed, a compressed air pressure pulse is delivered to the piston pressure line to propel the piston mass down the inserter tip. When the piston mass strikes the tip, it transfers some of its momentum to the tip, which pushes the array into the cortex in less than 1ms. To control the depth of UOA insertion, a spacer of desired length can be interposed between the piston and inserter tip. The spacer mechanically limits the travel distance of the inserter tip; two spacers are provided with the inserter, one for devices with 1.0 mm shank length, the other for 1.5 mm shank length devices. We used a 1 mm spacer for UOAs with >1 mm shank length, thus achieving partial insertion. **(C)** Exploded diagram of the main components of the inserter and their assembly. The device can be quickly and easily dis/re-assembled with spacers of different size for each UOA implantation (within a single surgery or across surgeries).

Supporting Figure 2. Statistical comparisons of GFAP immunoreactivity across cortical depths/layers in response to UOA insertion.

(A) Effect of insertion. For each UOA shank diameter group (Left, Middle and Right bar graphs) the raw Integrated Density of GFAP expression (expressed as Relative Fluorescent Units, RFUs, see Experimental Methods) for smooth/round (*s*) and rough/sharp (*r*) UOAs in different layers is statistically compared to each respective control (*ctrl*), using the Mann-Whitney test or t-test, as appropriate. *, **, ***, **** indicate statistical significance at the <0.05, <0.01, <0.001 and <0.0001 level, respectively. **(B)** Effect of shank diameter**.** For smooth/round and rough/sharp UOAs (Left and Right bar graphs) Integrated Density of GFAP expression is statistically compared across UOAs of different shank diameter in different layers, using the Kruskal-Wallis test or one-way ANOVA, as appropriate, corrected for multiple comparisons. Other conventions are as in (A). **(C)** Effect of geometry. For each UOA shank diameter group (Left, Middle and Right bar graphs) Integrated Density of GFAP expression is statistically compared across the two different geometries, smooth/round (*s*) and rough/sharp (*r*) UOAs in different layers, using the Mann-Whitney test or t-test, as appropriate. All statistical comparisons were conducted using biological replicates (Total n = 16 array insertions), with group sizes as follows: 60µm (60s: n = 3; 60r: n = 2), 85 µm (85s: n = 3; 85r: n = 3), and 100 µm (100s: n = 3; 100r: n = 2).

Supporting Figure 3. Double-immunostaining for GFAP and Iba1 in the same sections.

(A) Micrographs of two coronal sections double immunostained for both GFAP and Iba1 at the site of implantation of a 60µm shank diameter smooth/round UOA (Left) and a 60µm rough/sharp UOA (Right). Conventions are as in **Fig. 3A**. Left and right panels show the same sections as in

Fig. 5A and **5B**, respectively, but here shown in both channels. **(B)** Same as in (A) but for two 85μm shank diameter UOAs. Left and right panels show the same sections as in **Figs. 3C,5C**, and **Figs. 3D,5D**, respectively, but here shown in both channels simultaneously. **(C)** Same as in (A) but for two 100μm shank diameter UOAs. Left and right panels show the same sections as in **Figs. 3E,5E**, and **Fig. 5F**, respectively, but here but here shown in both channels simultaneously. Scale bar: 500μm and valid for all panels

Supporting Figure 4. Statistical comparisons of Iba1 immunoreactivity across cortical depths/layers in response to UOA insertion.

(A) Effect of insertion. For each UOA shank diameter group (Left, Middle and Right bar graphs) the raw Integrated Density of Iba1 expression for smooth/round (*s*) and rough/sharp (*r*) UOAs in different layers is statistically compared to each respective control (*ctrl*), using the Mann-Whitney test or t-test, as appropriate. *, **, ***, **** indicate statistical significance at the <0.05, <0.01, <0.001 and <0.0001 level, respectively. **(B)** Effect of shank diameter**.** For smooth/round and rough/sharp UOAs (Left and Right bar graphs) Integrated Density of Iba1 expression is statistically compared across UOAs of different shank diameter in different layers, using the Kruskal-Wallis test or one-way ANOVA, as appropriate, corrected for multiple comparisons. Other conventions are as in (A). **(C)** Effect of geometry. For each UOA shank diameter group (Left, Middle and Right bar graphs) Integrated Density of Iba1 expression is statistically compared across the two different geometries, smooth/round (*s*) and rough/sharp (*r*) UOAs in different layers, using the Mann-Whitney test or t-test, as appropriate. All statistical comparisons were conducted using biological replicates (Total n = 16 array insertions), with group sizes as follows: 60μm (60s: n = 3; 60r: n = 2), 85 μm (85s: n = 3; 85r: n = 3), and 100 μm (100s: n = 3; 100r: n = 2).

Supporting Figure 5. Double-immunostaining for GFAP and NeuN in the same sections.

(A) Micrographs of two coronal sections double immunostained for both GFAP and NeuN at the site of implantation of a 60μm shank diameter smooth/round UOA (Left) and a 60μm rough/sharp UOA (Right). Conventions are as in **Fig. 3A**. Left and right panels show the same sections as in **Figs. 3A,7A** and **3B,7B**, respectively, but here shown in both channels. **(B)** Same as in (A) but for two 85μm shank diameter UOAs. Left and right panels show the same sections as in **Fig. 7C** and **7D**, respectively, but here shown in both channels. **(C)** Same as in (A) but for two 100μm shank diameter UOAs. Left and right panels show the same sections as in **Fig. 7E**, and **Figs. 3F**, **7F**, respectively, but here shown in both channels simultaneously. Scale bar: 500μm and valid for all panels.

Supporting Figure 6. Statistical comparisons of NeuN immunoreactivity across cortical depths/layers in response to UOA insertion.

(A) Effect of insertion. For each UOA shank diameter group (Left, Middle and Right bar graphs) the raw Integrated Density of NeuN expression for smooth/round (*s*) and rough/sharp (*r*) UOAs in different layers is statistically compared to each respective control (*ctrl*), using the Mann-Whitney test or t-test, as appropriate. *, **, ***, **** indicate statistical significance at the <0.05, <0.01,

<0.001 and <0.0001 level, respectively. **(B)** Effect of shank diameter**.** For smooth/round and rough/sharp UOAs (Left and Right bar graphs) Integrated Density of NeuN expression is statistically compared across UOAs of different shank diameter in different layers, using the Kruskal-Wallis test or one-way ANOVA, as appropriate, corrected for multiple comparisons. Other conventions are as in (A). **(C)** Effect of geometry. For each UOA shank diameter group (Left, Middle and Right bar graphs) Integrated Density of NeuN expression is statistically compared across the two different geometries, smooth/round (*s*) and rough/sharp (*r*) UOAs in different layers, using the Mann-Whitney test or t-test, as appropriate. All statistical comparisons were conducted using biological replicates (Total $n = 16$ array insertions), with group sizes as follows: 60μm (60s: $n = 3$; 60r: $n = 2$), 85 μm (85s: $n = 3$; 85r: $n = 3$), and 100 μm (100s: $n = 3$; 100r: $n = 2$).

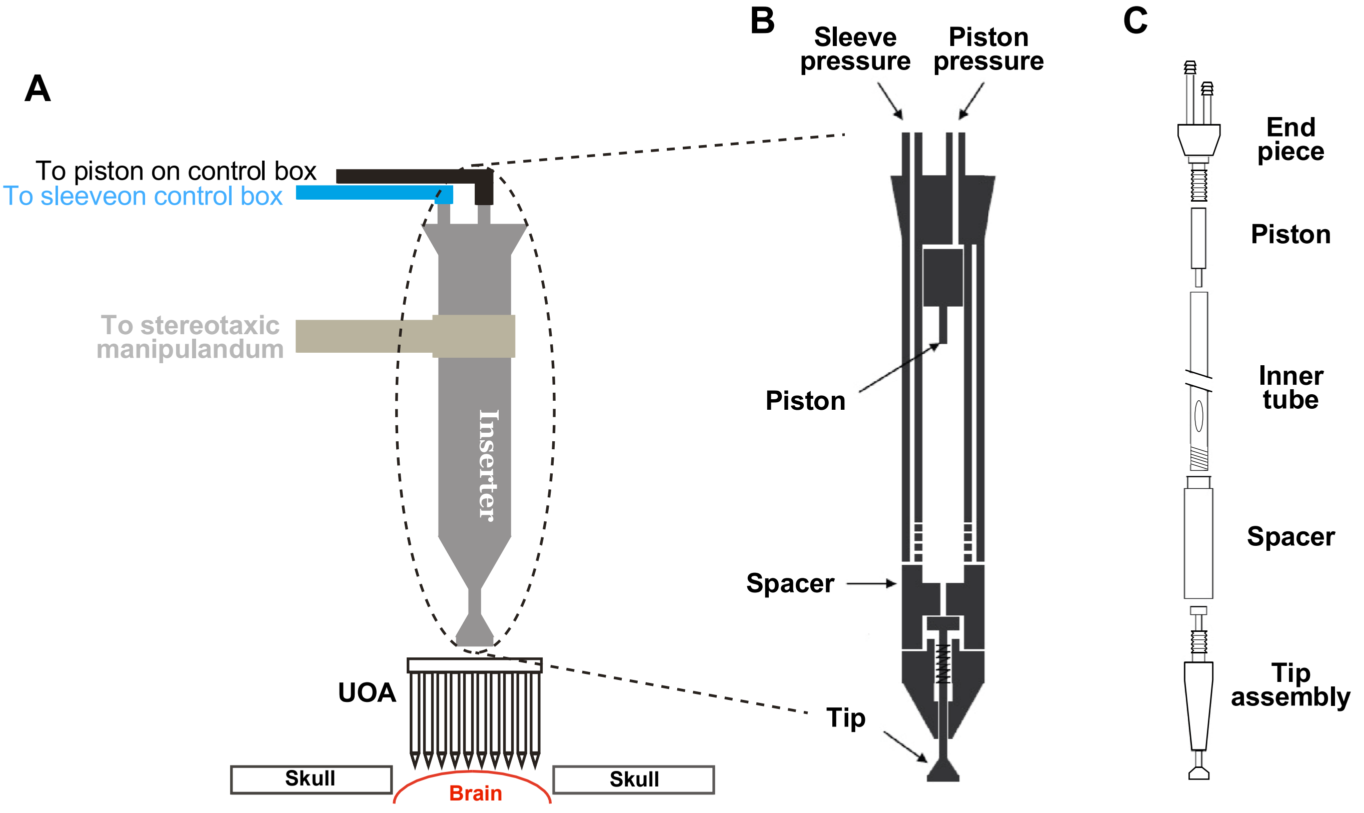

Supporting Figure 1

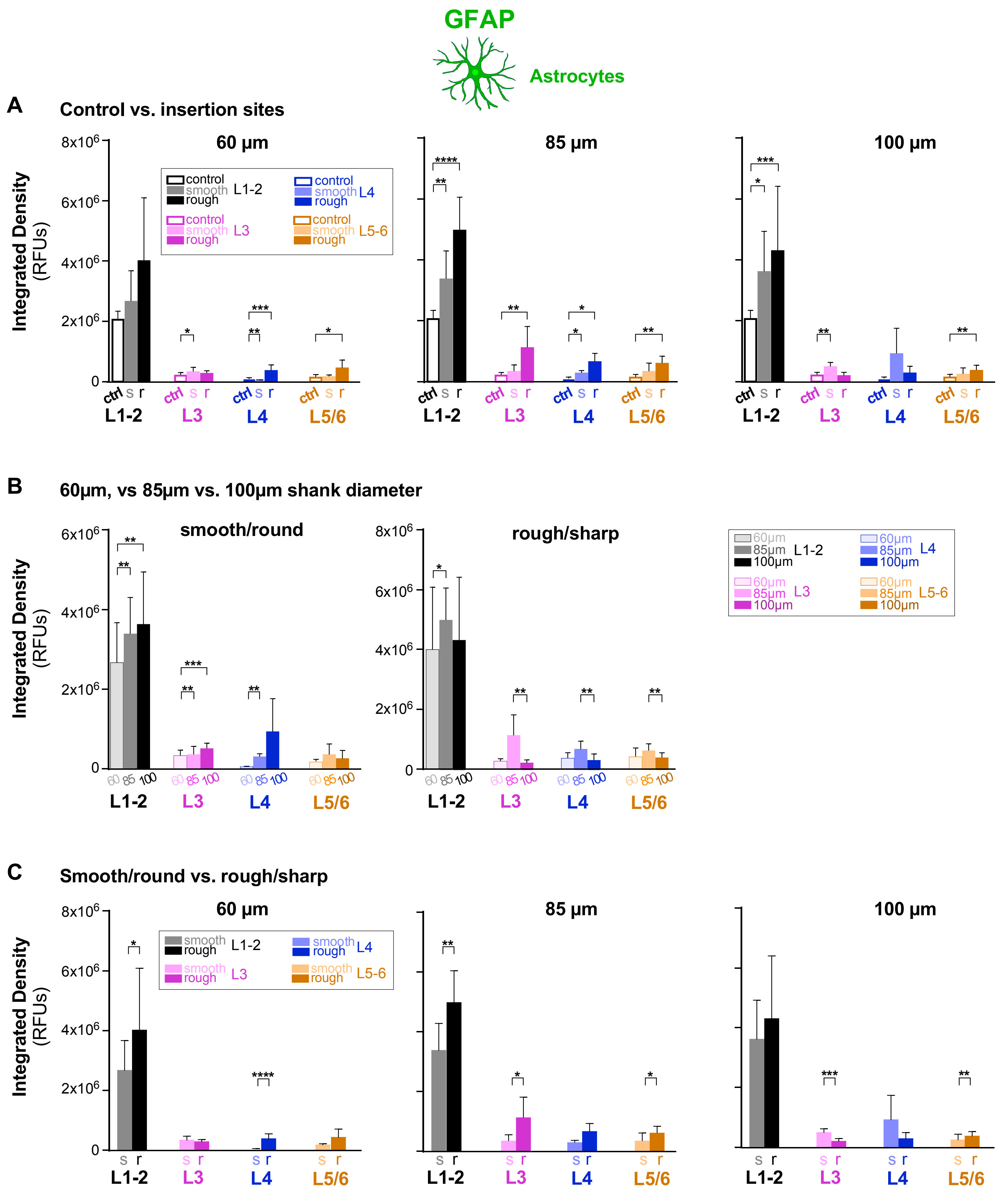

Supporting Figure 2

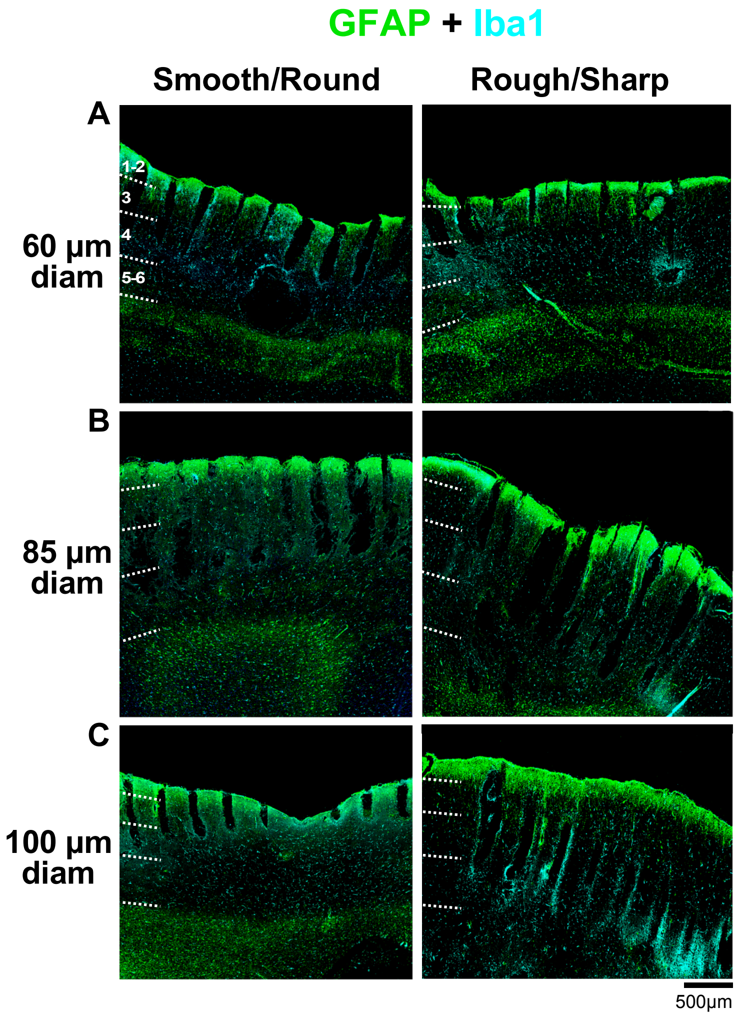

Suppporting Figure 3

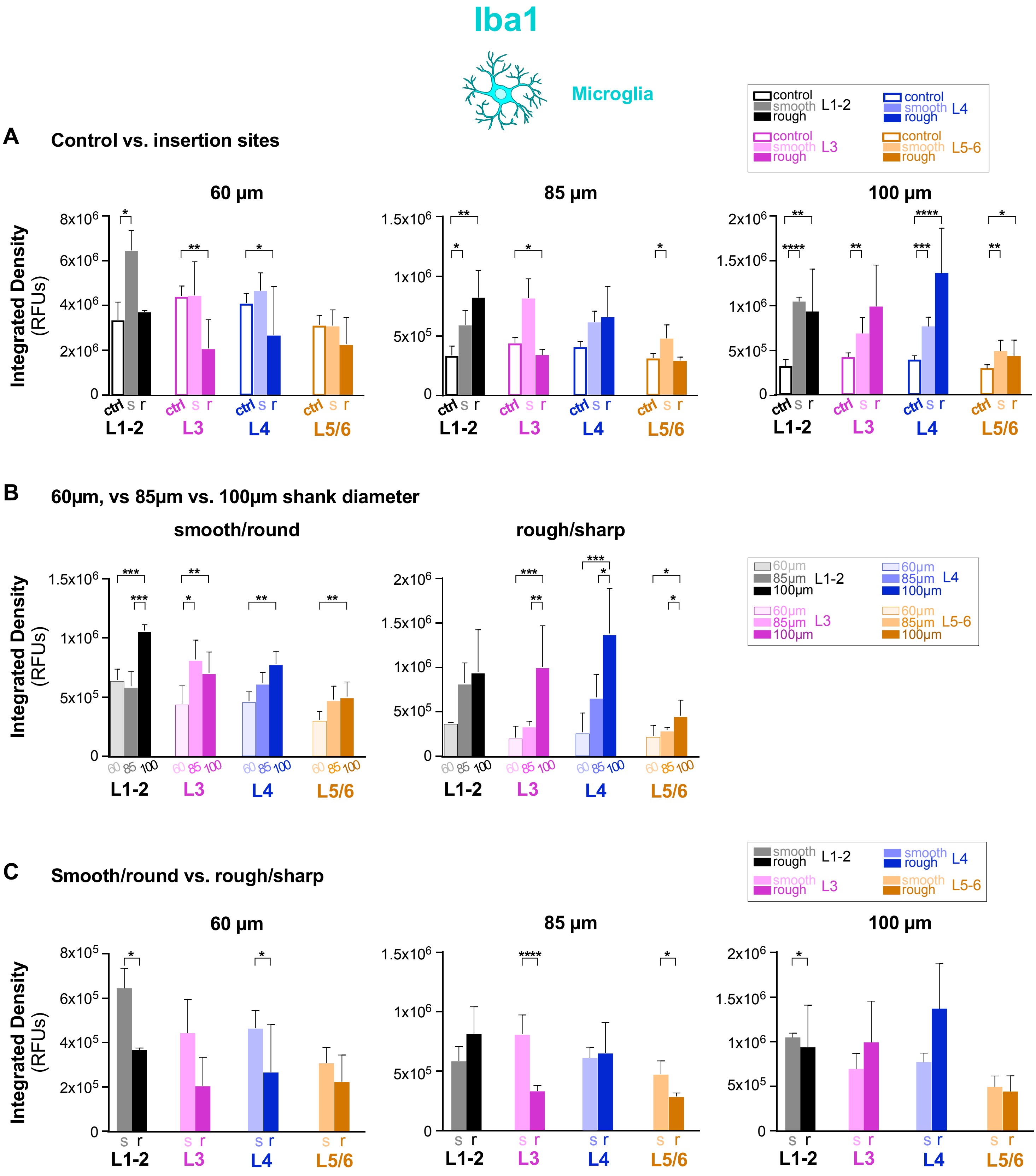

Supporting Figure 4

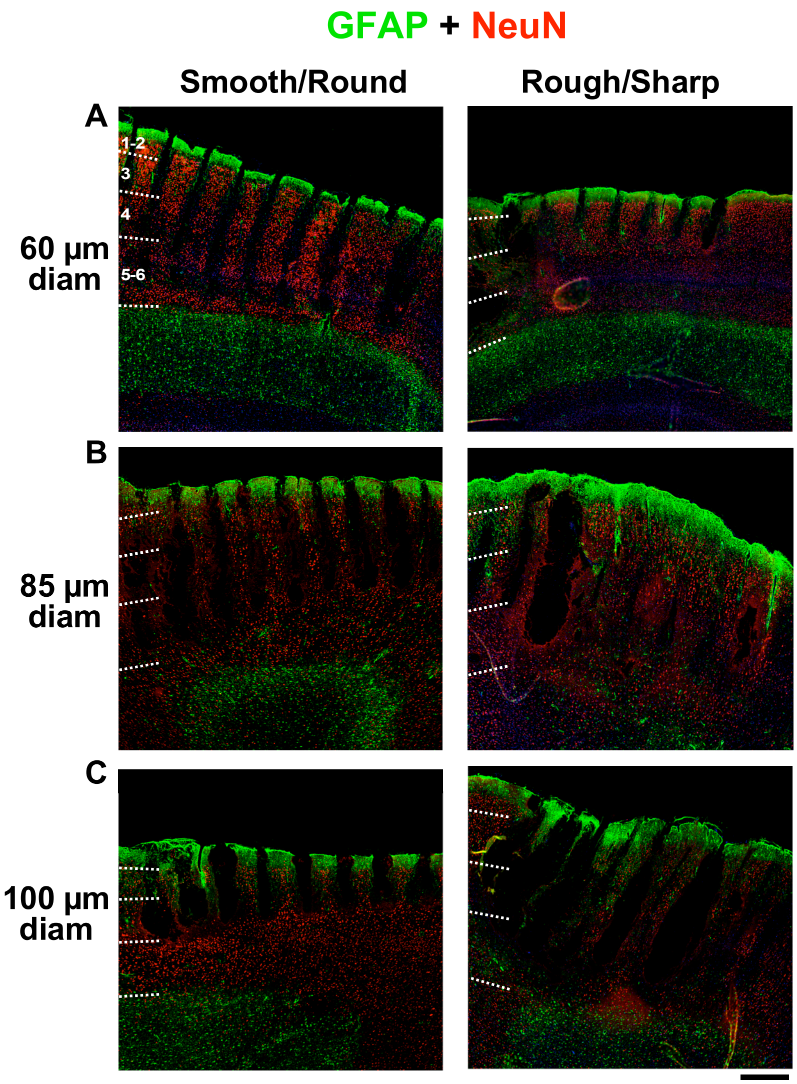

Supporting Figure 5

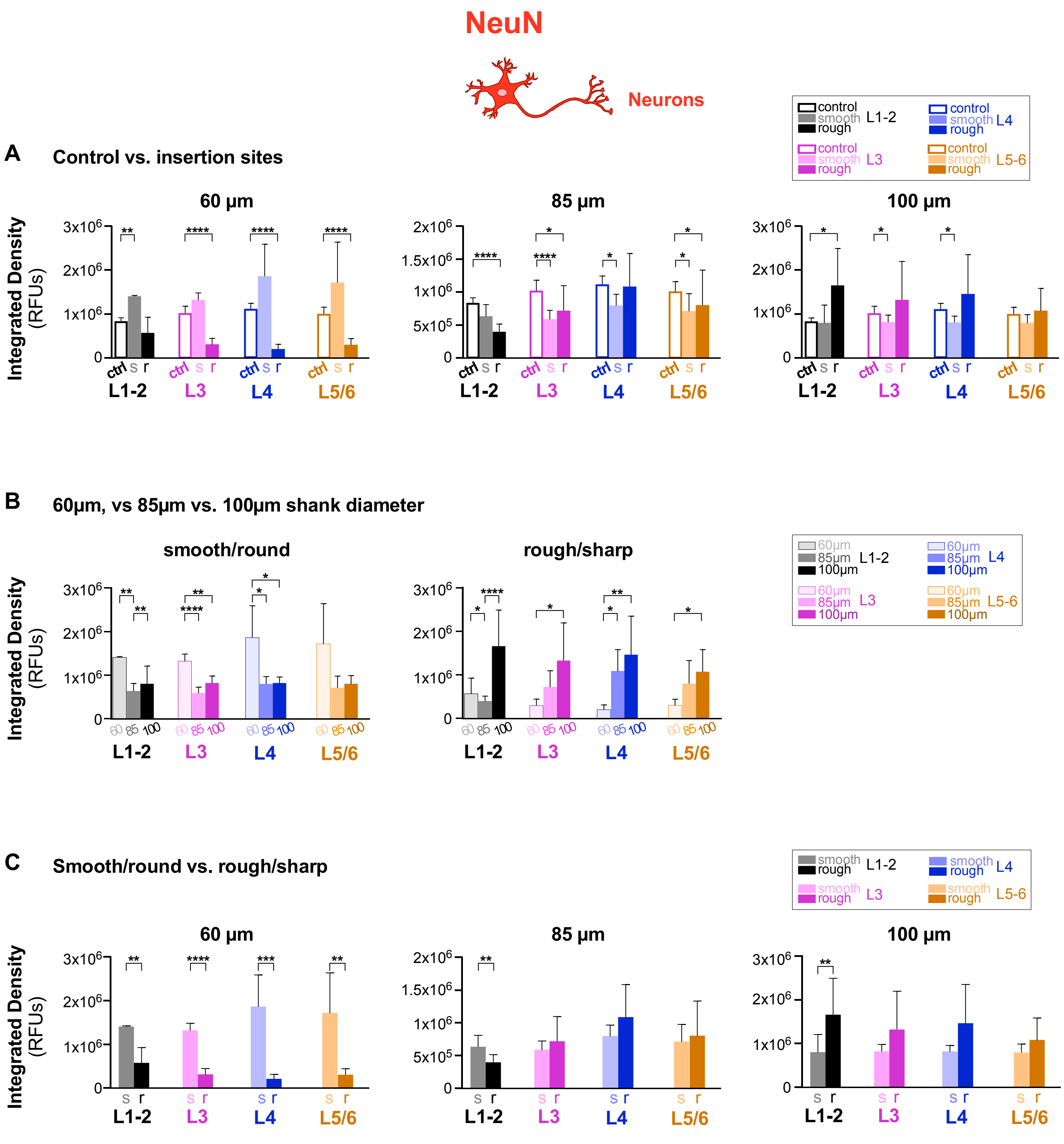

Supporting Figure 6
